# Editorial: Hemispheric asymmetries in the auditory domain, volume II

**DOI:** 10.3389/fnins.2023.1263317

**Published:** 2023-08-21

**Authors:** Nicole Angenstein, Alfredo Brancucci

**Affiliations:** ^1^Combinatorial NeuroImaging Core Facility, Leibniz Institute for Neurobiology, Magdeburg, Germany; ^2^Dipartimento di Scienze Motorie, Umane e della Salute, Università di Roma “Foro Italico”, Rome, Italy

**Keywords:** hemispheric asymmetries, functional lateralization, auditory perception, verbal cognition, musical cognition

Functional and structural asymmetries are present in many regions of the brain. The present Research Topic continues the description of studies on hemispheric asymmetries in the auditory domain (Westerhausen et al., 2014; Prete et al., 2016). The longest known is the left-lateralization of speech understanding (Della Penna et al., 2007; D'Anselmo et al., 2013). So far, many hemispheric asymmetries have been described for different kinds of auditory processing. Thereby, lateralization does not only depend on bottom-up stimulus-driven activity but also strongly on top-down influences (Brechmann and Scheich, 2005; Angenstein and Brechmann, 2013; Zatorre, 2022). For a given task, lateralization of processing is affected by task difficulty (Reiterer et al., 2005; Brechmann and Angenstein, 2019) but also other factors, such as aging (Stadler et al., 2023) and brain disorders (Ocklenburg et al., 2013; Prete et al., 2014; Serrallach et al., 2016). However, some questions remain about how asymmetries in the auditory domain are influenced by certain circumstances. Four articles in this Research Topic, using different methods (magnetic resonance imaging [MRI], magnetoencephalography [MEG], and behavioral measurements), investigated some of the open issues regarding the lateralization of auditory hallucinations (Mak et al.), effects of musical experience in combination with age (Bücher et al.), effects of tinnitus (Li et al.), and effects of the spatial location of sounds in the perception of emotional valence (Grisendi et al.).

Mak et al. experimentally induced verbal auditory hallucinations in neurotypical individuals by Pavlovian audio-visual conditioning separately in the left and right ear. After repeated pairing of an auditory monaurally presented word in noise with the same word on screen, they determined the number of false positives when presenting only the noise and the visual word. The authors suggested that these kinds of hallucinations might be left-lateralized, i.e., more false positives in the right ear, as most people have left-lateralized speech processing. However, no differences in false positive rates, perceptual sensitivities, and response biases were observed between the left and right ear. The authors discuss this in the context of reduced lateralization of speech processing in people with schizophrenia and verbal hallucinations.

Bücher et al., in their MEG-study, investigated the co-activation of auditory cortex areas and the orbitofrontal cortex in a large cohort (*N* = 162) of musicians and non-musicians of different ages during passive listening to instrument sounds and harmonic complex tones. For the P1 latency and amplitude, they observed differences in dependence on age and musical experience between the hemispheres. In children with musical experience, the P1 latency in the left hemisphere was shorter than in non-musicians but no differences were observed in adolescents and adults. Furthermore, the P1 amplitude in the right hemisphere was smaller in musicians than in non-musicians in children, larger in adults, and not different in adolescents. In all age groups in both hemispheres, the associated co-activation of the orbitofrontal cortex was shorter in musicians than in non-musicians and coincided in musicians with the P1. This means that musical experience leads to a different interaction between primary and secondary auditory responses and orbitofrontal co-activation.

Li et al., using arterial spin labeling, investigated the lateralization of cerebral blood flow (CBF) in the auditory cortex of patients with tinnitus. They observed hemispheric differences in CBF for people with and without tinnitus, with a higher CBF in the left primary auditory cortex than in the right one and a higher CBF in the right secondary and associative auditory regions than in the left ones. However, in the secondary auditory cortex, the CBF in the tinnitus patients was less lateralized to the right than in people without tinnitus. CBF in the primary auditory cortex was higher in people with tinnitus than those without tinnitus. This was interpreted as abnormal neuronal activity that might cause tinnitus. They hypothesized that patients with a higher CBF in the left primary auditory cortex have more difficulties adapting to tinnitus because of a correlation between this measure and tinnitus severity.

In a high-field functional magnetic resonance (fMRI) study, Grisendi et al. investigated brain activity elicited by human vocalizations in comparison with other environmental sounds and their modulation through emotional valence, as well as the effect of their source localization with respect to the listener. As similarly known for other sensory modalities (Klasen et al., 2012), the results indicate that the investigated parameters, i.e., emotional valence, spatial origin, and type of sound (environmental vs. human), interact with auditory neural activity already at early stages of processing. Moreover, a left auditory space asymmetry has been observed for vocalizations with positive emotional valence, which was not accompanied by hemispheric asymmetries and involved bilateral primary auditory cortices. Voice areas were sensitive to emotional valence but not to sound source localization. These results shed new light on the field of auditory perception, particularly with regard to the processing of spatial cues and emotional factors. The preponderance of positively laden emotional vocalizations in the left space certainly needs further investigation but offers a new picture of brain functioning that goes beyond the well-known hemispheric asymmetries in the auditory domain.

The studies constituting the present volume indicate that auditory hemispheric asymmetries concern multifaceted subjects that range from auditory pathology to ordinary perception. In the literature, very different techniques have been used to investigate the different features of the two hemispheres with regard to auditory perception, including dichotic listening (Brancucci and San Martini, 1999, 2003), non-invasive brain stimulation (D'Anselmo et al., 2015; Prete et al., 2018), analysis of functional connectivity (Brancucci et al., 2005, 2008; Angenstein and Brechmann, 2017; Stadler et al., 2023), and functional neuroimaging (Brancucci et al., 2011, 2016; Angenstein et al., 2016). These approaches are allowing a deeper understanding of auditory function, as substantiated in the present volume with regard to the psychological and neural bases of tinnitus and auditory hallucinations, two very common psychopathologies for which several efforts have been made to search for an effective treatment. In parallel, a better understanding of the ordinary functioning of the auditory system, through analyses that investigate the neurocognitive bases of musical, verbal, and environmental sounds, lays a groundwork that can aid researchers from the multidisciplinary field of neurosciences to obtain an ever clearer picture of how our brain works.

## Author contributions

NA: Writing—original draft, Writing—review and editing. AB: Writing—original draft, Writing—review and editing.
